# EPISTASIS ASSOCIATION STUDY TO IDENTIFY NETWORK HUB GENES IN ALZHEIMER’S DISEASE AND RELATED DEMENTIAS

**DOI:** 10.1093/geroni/igad104.3306

**Published:** 2023-12-21

**Authors:** Luke Evans, Chandra Reynolds, Charles Hoeffer

**Affiliations:** University of Colorado Boulder, Boulder, Colorado, United States; University of Colorado Boulder, Boulder, Colorado, United States; University of Colorado Boulder, Boulder, Colorado, United States

## Abstract

Despite recent advances in understanding ADRD genetic risk, the vast majority of the genetic variance of AD/ADRD risk remains unexplained by identified loci such as APOE or the polygenic signal. One unexplored reservoir of genetic effects is interactions among genes (epistasis), which could identify key and novel molecular pathways, networks, and specific cell types in which the ADRD genetic risk is manifest. We tested all pairwise gene-gene interactions using our recently-developed transcriptome-wide interaction study method on ADRD in the UKBiobank. While no individual epistatic association was genome-wide significant (p< 2.56e-10), several approached significance (p< 3e-8). More importantly, we identified three core genes with >10 suggestive epistatic interactions. These include genes with some limited, prior evidence of a role in ADRD etiology, but none that have been identified in single-locus GWAS. In follow-up enrichment analyses of neuronal annotations underlying the interaction associations, we found genes intolerant to protein truncating mutations (PI), and those expressed specifically in endothelial cells, excitatory neurons in the prefrontal cortex , neuronal stem cells, and PI excitatory dentate gyrus cells to be nominally enriched (p< 0.05) in interaction associations with ADRD using prefrontal cortex imputed expression. Together, these suggest that we can uncover novel aspects of AD biology through these interaction associations and identify genes that may play a key role in integrating signals of AD risk. We anticipate identifying additional core genes and pathways as we integrate other larger and more diverse datasets.

